# Speckle tracking technology and investigation of risk factors for premature ventricular contraction-induced cardiomyopathy

**DOI:** 10.3389/fcvm.2025.1675906

**Published:** 2025-09-30

**Authors:** Qiao Ma, Beibei Zou, Yangkai Shi, Qianqian He, Muhua Zhang, Chao Feng

**Affiliations:** ^1^ Department of Nursing, The Fourth Affiliated Hospital of School of Medicine, and International School of Medicine, International Institutes of Medicine, Zhejiang University, Yiwu, China; ^2^ Department of Cardiology, The Fourth Affiliated Hospital of School of Medicine, International School of Medicine, International Institutes of Medicine, Zhejiang University, Yiwu, China

**Keywords:** speckle tracking echocardiography, premature ventricular contractions, PVCCM, ventricular remodeling, risk factors

## Abstract

**Background:**

Premature ventricular contractions (PVCs) are increasingly recognized as a potentially reversible cause of cardiomyopathy, termed PVC-induced cardiomyopathy (PVCCM). Left ventricular ejection fraction (LVEF) is commonly used for diagnosis, but it lacks sensitivity for detecting early myocardial dysfunction. This study aimed to evaluate the diagnostic utility of speckle tracking echocardiography (STE)-derived strain parameters in patients with frequent PVCs and to identify associated risk factors for early myocardial impairment.

**Methods:**

A total of 258 patients with monomorphic PVCs and a PVC burden >5% on 24 h Holter monitoring were enrolled, along with 80 age- and sex-matched healthy controls. Conventional echocardiographic parameters, global longitudinal strain (GLS), and global circumferential strain (GCS) were measured. Linear regression analyses were performed to identify independent predictors of impaired strain. Subgroup analyses were conducted based on comorbidities and electrophysiological features.

**Results:**

Despite comparable LVEF between the PVC and control groups, GLS and GCS showed attenuated magnitude in PVC patients (*P* < 0.001), indicating subclinical dysfunction. Regression analysis revealed that asymptomatic PVCs, paired/interpolated PVCs, wide QRS duration (≥150 ms), and higher PVC burden were significantly associated with decreased GLS and GCS magnitude. GLS and GCS showed strong inverse correlations with LVEF, particularly in patients with hypertension or prolonged QRS duration.

**Conclusion:**

STE-derived strain parameters (GLS, GCS) are more sensitive than LVEF in detecting early myocardial dysfunction in patients with frequent PVCs. Specific electrocardiographic features may help identify individuals at higher risk of strain abnormalities and could inform earlier monitoring or targeted evaluation; prospective studies are needed to establish whether intervention based on these markers prevents progression to overt PVCCM.

## 1 Introduction

Premature ventricular contraction (PVC) refers to ventricular premature contractions originating from ectopic foci located below the His bundle and its branches due to premature depolarization of ventricular myocardium (1). Increasing evidence suggests that PVCs are associated with cardiac dysfunction. Frequent PVCs can lead to ventricular remodeling and may eventually progress to cardiomyopathy and heart failure (2, 3). This type of cardiomyopathy has been termed premature ventricular contraction-induced cardiomyopathy (PVCCM) (1, 4). Although no standardized definition has been established in current guidelines, most studies define PVCCM as a condition in which the left ventricular ejection fraction (LVEF) is ≤50% (5). However, in the early stages of the disease, ventricular remodeling or mild systolic dysfunction may already be present despite a preserved LVEF (6). Therefore, identifying an appropriate marker for the early prediction of PVCCM is crucial. In this study, we adopted a PVC burden threshold of >5% for patient selection. This cutoff has been supported by several clinical studies, which have demonstrated its association with adverse cardiac events (7, 8), myocardial remodeling (9), and early markers of myocardial fibrosis (10), and it is widely considered a clinically meaningful threshold.

Speckle Tracking Echocardiography (STE) tracks myocardial tissue motion by identifying acoustic speckles within the myocardium (11, 12). Using specialized analytical software, STE analyzes myocardial displacement at various points within the region of interest during the cardiac cycle, ultimately calculating myocardial strain. Based on the direction of deformation, myocardial strain can be classified into global longitudinal strain (GLS), global radial strain (GRS), and global circumferential strain (GCS) (13). Multiple clinical studies have demonstrated a strong correlation between strain parameters measured by STE and LVEF in patients with cardiomyopathy. Moreover, STE can detect strain abnormalities in cardiomyopathy or coronary artery disease (CAD) patients with preserved LVEF, highlighting its potential for early detection of myocardial dysfunction (13–17).

Currently, the diagnosis of PVCCM largely relies on retrospective analysis and exclusion diagnosis. However, in the early stages of the disease, patients may already exhibit ventricular remodeling or mild systolic dysfunction despite a normal LVEF (18). Predicting cardiomyopathy remains a significant challenge in the field of cardiovascular diseases. This study aims to enroll patients with frequent PVCs and use conventional echocardiography combined with speckle tracking analysis to assess myocardial strain, enabling the early diagnosis of PVCCM. Additionally, we seek to identify risk factors for PVCCM, with the ultimate goal of facilitating timely and effective interventions.

## 2 Materials and methods

### 2.1 Study design and populations

This study was a cross-sectional analysis conducted between June 2021 and December 2022 at the Fourth Affiliated Hospital of Zhejiang University School of Medicine. Patients with frequent PVCs were enrolled based on the following inclusion criteria: (1) Age between 18 and 80 years; (2) Completion of 24 h Holter monitoring, demonstrating a PVC burden >5% within 24 h (based on a single baseline recording rather than cumulative monitoring); (3) Predominantly monomorphic PVCs, with the primary PVC morphology accounting for >90% of total PVCs; (4) First-time diagnosis of frequent PVCs, with no prior antiarrhythmic therapy or electrophysiological interventions. The >5% PVC burden was derived exclusively from the baseline Holter monitoring, independent of unmonitored or asymptomatic periods, ensuring that the burden assessment reflected the initial presentation. Written informed consent was obtained from all participants. The exclusion criteria were as follows: (1) Coexisting arrhythmias with a burden exceeding 10% of total beats on 24 h Holter monitoring; (2) Multifocal PVCs; (3) Presence of structural heart disease; (4) Frequent PVCs related to secondary conditions, including infections, anemia, hyperthyroidism, electrolyte imbalances, severe trauma, or perioperative stress; (5) Active malignancies; (6) History of psychiatric disorders or ongoing use of psychotropic medications. Healthy Control Group: A total of 80 healthy individuals undergoing routine medical examinations at the hospital during the same period were recruited as controls. The control group was matched to the PVC group by age and sex. Participants had no significant medical history, no family history of cardiomyopathy, and presented with normal physical examinations, biochemical tests, electrocardiography (ECG), and echocardiographic findings. This study was approved by the Ethics Committee of the Fourth Affiliated Hospital of Zhejiang University School of Medicine. Written informed consent was obtained from all participants.

### 2.2 Data collection and definitions

Demographic and clinical data were collected, including: (1) gender, age, height, weight, and body mass index (BMI); (2) Smoking and drinking history; (3) Presence of hypertension or diabetes mellitus; (4) Presence of clinical symptoms (e.g., palpitations, skipped beats, or tachycardia sensations).

Electrocardiographic Recordings A 12-lead surface electrocardiogram (ECG) and a simultaneous 24 h Holter ECG were recorded to assess PVC episodes. The presence of the following PVC-related characteristics was analyzed: interpolated PVC, paired PVC, and NSVT. The PVC burden was calculated as the ratio of total PVC beats to total heartbeats over 24 h. Based on the hourly PVC count and the corresponding mean heart rate, patients were categorized into three rhythm-dependent PVC subtypes (19): fast heart rate-dependent PVC (F-HR-PVC), slow heart rate-dependent PVC (S-HR-PVC), and independent heart rate PVC (I-HR-PVC).

For 12-lead ECG analysis, the following PVC-related parameters were manually measured using calipers: QRS duration, QT interval, coupling interval, and compensatory pause. Additionally, the coupling interval index and compensatory pause index were calculated. The PVC site was determined by two experienced electrophysiologists based on prior knowledge and clinical expertise and classified as originating from right ventricle and originating from left ventricle (20). Furthermore, based on the inferior lead QRS morphology, PVCs were categorized as outflow tract origin PVCs or non-outflow tract origin PVCs (21).

Patients were positioned in the left lateral decubitus position, and routine echocardiographic parameters were collected. Left ventricular end-diastolic volume (LVEDV), left ventricular end-systolic volume (LVESV), and left ventricular ejection fraction (LVEF) were measured using the biplane Simpson's method. Image acquisition followed EACVI/ASE guidelines (22), with the transducer frequency set at 2–4 MHz, sector width optimized to maintain temporal resolution >50 Hz, and each view obtained during breath-hold to minimize motion artifacts.

High-frame-rate echocardiography (≥250 frames/s) was employed, with frame rates adjusted according to heart rate to ensure at least 20 frames per cardiac cycle for reliable strain analysis (23–26). Real-time speckle tracking imaging data were obtained from apical four-chamber, two-chamber, and three-chamber views, with 4–5 cardiac cycles stored digitally for offline analysis. Strain measurements were performed using the EchoPAC workstation (2D-Strain, Q-analysis) with standardized settings and automatic quality control.

**Figure 1:**
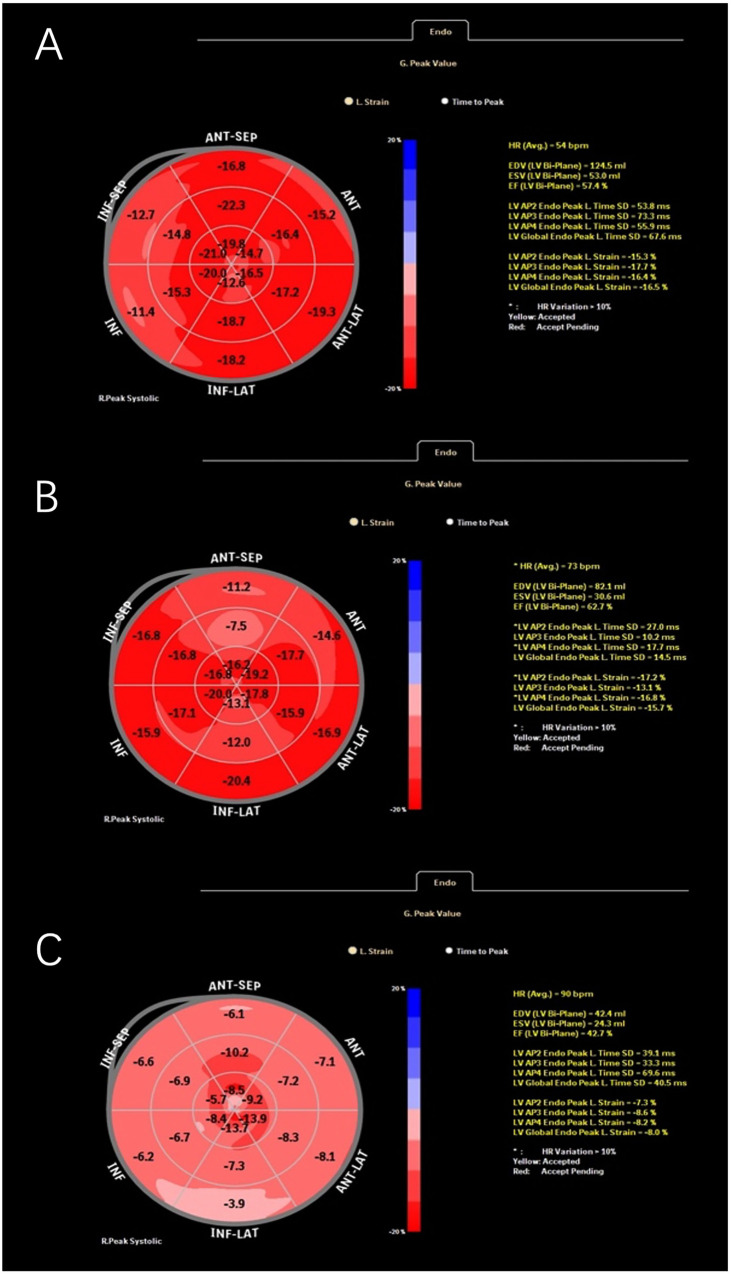
Representative global longitudinal strain (GLS) curves obtained from speckle-tracking echocardiography in different patients: ventricular premature patient 1 **(A)**, ventricular premature patient 2 **(B)**, and a patient with myocarditis **(C)**.

For strain analysis, the endocardial border was manually traced and the region of interest selected. The software then automatically tracked myocardial strain, providing segmental global longitudinal strain (GLS) and global circumferential strain (GCS) values. Representative examples of GLS strain curves are shown in Figure 1. GLS and GCS are reported as negative percentages. Less negative values indicate impaired myocardial deformation (reduced magnitude), whereas more negative values indicate greater deformation (increased magnitude) (27, 28). All stored loops were reviewed for adequate frame rate (>50 fps) and tracking quality before analysis. Frames with significant out-of-plane motion or dropout were excluded, and the motion estimation algorithm incorporated co-attention mechanisms to resolve inter-frame variations (29). Manual correction was performed when automated tracking failed, and inter-frame interpolation was applied when necessary to maintain analysis continuity (30). Although we excluded image frames immediately following PVCs to minimize post-extrasystolic effects, brief alterations in myocardial relaxation due to calcium handling or NSVT episodes may still influence strain measurements.

All image loops were reviewed for frame rate and tracking quality before analysis, and segments with dropout or poor tracking were excluded. Each strain measurement was performed by a single experienced echocardiographer, repeated three times, and averaged for analysis. Accordingly, the reproducibility assessed in this study reflects intraobserver variability rather than interobserver variability. Previous reports have demonstrated excellent intraobserver reproducibility of speckle tracking (intraclass correlation coefficient up to 0.98) (31). This protocol ensured reproducibility through standardized high-frame-rate acquisition, automated tracking with manual verification, repeated measurements, and adherence to international guidelines for strain imaging (22, 32, 33).

### 2.3 Statistical analysis

Continuous variables were expressed as mean ± standard deviation (SD) for normally distributed data or as median with interquartile range (IQR) for non-normally distributed data. Group comparisons were conducted using the Student's *t*-test for normally distributed continuous variables. Categorical variables were presented as frequencies (percentages) and compared using the Chi-square (*χ*^2^) test. The correlations between GLS, GCS, and other continuous variables were assessed using Spearman correlation analysis. Comparisons between GLS, GCS, and categorical variables were conducted using *t*-tests or one-way ANOVA. To identify independent factors influencing GLS and GCS, univariate and multivariate bidirectional stepwise linear regression analyses were performed. Variables with *P* < 0.1 in univariate analysis were included in the multivariate regression model. Based on the results of multivariate linear regression, trend analysis and regression modeling were conducted to explore the relationship between GLS, GCS, and LVEF. Model 1 was the basic model without any covariate adjustment. Model 2 was adjusted for gender, age, and body mass index (BMI). Model 3 was further adjusted for gender, age, BMI, QRS duration, paired PVC, interpolated PVC and symptomatic PVC. Covariates were selected based on three considerations: (1) clinical relevance and prior evidence, whereby gender, age, and BMI are routinely adjusted for in cardiovascular studies; (2) results from separate multivariable regression analyses of GLS and GCS, in which predictors with significant associations were identified; and (3) overlapping positive predictors between GLS and GCS analyses were retained to construct the final models. To further ensure model stability, we assessed multicollinearity among covariates by calculating variance inflation factors (VIFs) and condition indices, with results summarized in Supplementary Table S1. Additionally, subgroup analyses were conducted based on the following factors: gender, presence of hypertension, presence of diabetes mellitus, smoking history, drinking history, PVC rhythm-dependent type, left or right ventricular origin, outflow tract or non-outflow tract origin, QRS duration group, presence of paired PVC, presence of interpolated PVC, presence of NSVT, and presence of clinical symptoms. Data processing and statistical analyses were performed using R (latest version) and Zstats v1.0 (https://www.zstats.net). All statistical tests were two-tailed, with *P* < 0.05 considered statistically significant.

## 3 Results

### 3.1 Baseline characteristics

From June 2021 to December 2022, a total of 258 patients with PVCs were enrolled in this study after applying the inclusion and exclusion criteria, forming the PVC group. Additionally, 80 age- and gender-matched healthy individuals who underwent routine health examinations were included as the control group. The differences in baseline characteristics and cardiac function parameters between the PVC group and the control group are summarized in Table 1. There were no statistically significant differences between the two groups in terms of age, BMI, gender, prevalence of hypertension or diabetes mellitus, smoking history, or drinking history. Comparisons of cardiac function parameters revealed that LVEF was not significantly different between the two groups (*P* = 0.150). However, both GLS and GCS showed significant differences between the groups (*P* < 0.001), suggesting early myocardial dysfunction despite preserved LVEF.

**Table 1:** Baseline characteristics and cardiac function indexes between PVC group and control group.

Variables	Total (*n* = 338)	0 (*n* = 80)	1 (*n* = 258)	Statistic	*P*
Age, Mean ± SD	45.21 ± 14.48	45.58 ± 13.22	45.09 ± 14.87	*t* = 0.26	0.795
BMI, Mean ± SD	25.41 ± 3.32	25.23 ± 2.90	25.46 ± 3.44	*t* = −0.55	0.584
LVEF, Mean ± SD	61.50 ± 5.25	62.16 ± 4.40	61.29 ± 5.48	*t* = 1.45	0.150
GLS, Mean ± SD	−15.71 ± 2.54	−18.70 ± 2.06	−14.78 ± 1.88	*t* = −15.92	<.001
GCS, Mean ± SD	−15.68 ± 2.59	−18.50 ± 2.09	−14.80 ± 2.04	*t* = −14.06	<.001
Gender (*n*, %)				*χ*^2^ = 0.55	0.458
Female	161 (47.63)	41 (51.25)	120 (46.51)		
Male	177 (52.37)	39 (48.75)	138 (53.49)		
Hypertension (*n*, %)				*χ*^2^ = 0.52	0.472
No	256 (75.74)	63 (78.75)	193 (74.81)		
Yes	82 (24.26)	17 (21.25)	65 (25.19)		
DM (*n*, %)				*χ*^2^ = 1.30	0.254
No	296 (87.57)	73 (91.25)	223 (86.43)		
Yes	42 (12.43)	7 (8.75)	35 (13.57)		
Smoking (*n*, %)				*χ*^2^ = 0.32	0.571
No	267 (78.99)	65 (81.25)	202 (78.29)		
Yes	71 (21.01)	15 (18.75)	56 (21.71)		
Drinking (*n*, %)				*χ*^2^ = 0.22	0.636
No	225 (66.57)	55 (68.75)	170 (65.89)		
Yes	113 (33.43)	25 (31.25)	88 (34.11)		

*t,*: *t*-test, χ^2^, Chi-square test; SD, standard deviation.

### 3.2 Electrocardiographic and Holter characteristics in PVC patients

In the enrolled PVC patients, the mean QT interval, QRS duration, coupling interval, and compensatory pause were 491.55 ms, 138.11 ms, 452.12 ms, and 983.57 ms, respectively (Table 2). Among them, 16.7% of patients had a QRS duration ≥150 ms. Most PVCs originated from the right ventricle (61.6%) and outflow tract (67.1%) (Table 2). The mean PVC burden was 10.94%, with 36.4% of patients experiencing paired PVC, 34.5% exhibiting interpolated PVC, and 17.8% having NSVT. The majority of patients (58.5%) had F-HR-PVC, whereas 25.6% had I-HR-PVCs, and only 15.9% exhibited S-HR-PVC (Table 2). Additionally, 55.8% of PVC patients reported symptoms such as palpitations, tachycardia, or a sensation of skipped beats (Table 2).

**Table 2:** Electrocardiographic and holter characteristics in PVC group.

Variables	Values
Electrocardiographic Characteristics	
QT interval, ms	491.55 ± 24.33
QRS duration, ms	138.11 ± 11.46
QRS ≥ 150 ms, % (*n*)	16.7 (43)
Coupling interval, ms	452.12 ± 38.15
Compensatory pause, ms	983.57 ± 83.62
Coupling interval index	0.63 ± 0.06
Compensatory pause index	1.37 ± 0.08
Originating from right ventricle, % (*n*)	61.6 (159)
Originating from outflow tract, % (*n*)	67.1 (173)
Holter Characteristics	
PVC burden, %	10.94 ± 5.41
Paired PVC, % (*n*)	36.4 (94)
Interpolated PVC, % (*n*)	34.5 (89)
Non-sustained ventricular tachycardia, % (*n*)	17.8 (46)
PVC course	
Fast-HR-dependent PVC, % (*n*)	58.5 (151)
Slow-HR-dependent PVC, % (*n*)	15.9 (41)
Independent-HR-PVC, % (*n*)	25.6 (66)
Symptomatic PVC, % (*n*)	55.8 (144)

### 3.3 GLS, GCS, and their associations with baseline and electrocardiographic parameters

The mean age of the included patients was 45.2 years, with an average BMI of 25.4 kg/m^2^ (Table 1). GLS was significantly correlated with age (*R* = 0.178, *P* = 0.004), BMI (*R* = 0.192, *P* = 0.002), hypertension (*P* = 0.001), smoking (*P* = 0.043), and PVC burden (*R* = 0.186, *P* = 0.003), whereas GCS was mainly associated with PVC burden (*R* = 0.132, *P* = 0.035). Patients with asymptomatic PVCs had attenuated GLS and GCS magnitude compared with symptomatic patients (both *P* < 0.05). In addition, patients with wide QRS exhibited significantly impaired GLS (*P* = 0.011). Specific PVC morphologies—including paired PVCs, interpolated PVCs, and NSVT—were also significantly associated with impaired GLS and GCS (all *P* < 0.05). Other baseline characteristics and electrophysiological parameters showed no significant associations with strain indices. Detailed results are presented in Supplementary Tables S2, S3.

### 3.4 Independent correlates of GLS and GCS

In univariate analyses, age, BMI, hypertension, smoking, symptomatic PVCs, PVC burden, wide QRS, paired PVCs, interpolated PVCs, NSVT, and LVEF were significantly associated with attenuated GLS magnitude (all *P* < 0.05). In multivariable regression, symptomatic PVCs (*β* = −0.99, *P* < 0.001), wide QRS (*β* = 0.97, *P* < 0.001), paired PVCs, interpolated PVCs, PVC burden, and LVEF remained independent correlates of GLS impairment. For GCS, univariate regression identified associations with symptomatic PVCs, paired PVCs, interpolated PVCs, NSVT, PVC burden, and LVEF (all *P* < 0.05). In multivariable models, symptomatic PVCs (*β* = −0.70, *P* = 0.004), wide QRS (*β* = 0.84, *P* = 0.009), paired PVCs, interpolated PVCs, and LVEF were independent predictors of reduced GCS magnitude. Notably, symptomatic PVCs were inversely associated with impaired strain, indicating that asymptomatic PVCs were relatively more detrimental. Comprehensive regression outputs are provided in Supplementary Tables S4, S5.

### 3.5 Linear regression modeling analysis of GLS, GCS, and LVEF

Linear regression modeling showed a consistent association between lower LVEF and attenuated strain magnitude. In the unadjusted model, the regression coefficient (*β* = −0.06, 95% CI: −0.10 to −0.02, *P* = 0.004) indicates that a 1% lower LVEF corresponds to a 0.06-unit increase in GLS value, reflecting reduced GLS magnitude (Table 3). After adjusting for gender, age, and BMI, the negative correlation remained significant (*β* = −0.06, 95% CI: −0.10 to −0.01, *P* = 0.008) (Table 3). Further adjustments for QRS duration, paired PVC, interpolated PVC and symptomatic PVC still showed a significant correlation (*β* = −0.04, 95% CI: −0.08 to −0.01, *P* = 0.010), though the regression coefficient slightly decreased, suggesting that confounding factors influenced the relationship but did not alter the overall trend (Table 3). Similarly, lower LVEF was associated with attenuated GCS magnitude (unadjusted *β* = −0.08, 95% CI: −0.12 to −0.04, *P* < 0.001; Model 2 *β* = −0.07, 95% CI: −0.12 to −0.03, *P* = 0.002; Model 3 *β* = −0.06, 95% CI: −0.11 to −0.02, *P* = 0.005), indicating that lower LVEF corresponds to attenuated GCS magnitude (Table 4). Overall, the negative correlation between LVEF and GCS remained statistically significant across all models, with no change in the trend after adjusting for confounders. Across all models, the negative associations between LVEF and both GLS and GCS remained robust after progressive adjustment. Multicollinearity diagnostics showed that all covariates had VIFs <2.0, and no concerning condition indices were detected, indicating that collinearity was not a concern (Supplementary Table S1).

**Table 3:** Linear regression modeling analysis of GLS and LVEF.

Variables	Model1		Model2		Model3	
	*β* (95%CI)	*P*	β (95%CI)	*P*	β (95%CI)	*P*
LVEF	−0.06 (−0.10 to −0.02)	**0** **.** **004**	−0.06 (−0.10 to −0.01)	**0** **.** **008**	−0.04 (−0.08 to −0.01)	**0** **.** **039**

CI: Confidence Interval.

Model1: Crude.

Model2: Adjust: Gender, Age, BMI.

Model3: Adjust: Gender, Age, BMI, QRS duration, Paired PVC, Interpolated PVC, Symptomatic PVC.

Bold values indicate *P* < 0.05.

**Table 4:** Linear regression modeling analysis of GCS and LVEF.

Variables	Model1		Model2		Model3	
	*β* (95%CI)	*P*	*β* (95%CI)	*P*	*β* (95%CI)	*P*
LVEF	−0.08 (−0.12 to −0.04)	**<** **.** **001**	−0.07 (−0.12 to −0.03)	**0** **.** **002**	−0.06 (−0.11 to −0.02)	**0** **.** **005**

CI: Confidence Interval.

Model1: Crude.

Model2: Adjust: Gender, Age, BMI.

Model3: Adjust: Gender, Age, BMI, QRS duration, Paired PVC, Interpolated PVC, Symptomatic PVC.

Bold values indicate *P* < 0.05.

### 3.6 Subgroup analysis of the relationship between GLS, GCS, and LVEF

A subgroup analysis was performed based on gender, presence of hypertension, diabetes mellitus, smoking history, alcohol consumption, arrhythmic classification, PVC site, QRS group, presence of paired PVC, interpolated PVC, NSVT, and symptomatic status. Results showed that LVEF and GLS were significantly negatively correlated across all patients (*β* = −0.53; 95% CI: −0.88 to −0.18; *P* = 0.004), with consistent trends across all subgroups (Table 5). Similarly, LVEF and GCS were also significantly negatively correlated (*β* = −0.57; 95% CI: −0.89 to −0.25; *P* = 0.001). A nominally significant interaction effect was observed in patients with hypertension (*P* = 0.037) and in those with wide QRS (*P* = 0.003) (Table 6). Interaction Analysis: In hypertensive patients, the negative correlation between LVEF and GCS was more pronounced (*β* = −1.21; 95% CI: −1.87 to −0.54; *P* < 0.001). In patients with wide QRS, the negative correlation was even stronger (*β* = −1.72; 95% CI: −2.56 to −0.89; *P* < 0.001) (Table 6). For all other subgroups, the negative correlation between LVEF and GCS remained consistent.

**Table 5:** Subgroup analysis of the relationship between GLS and LVEF.

Variables	*n* (%)	*β* (95%CI)	*P*	*P* for interaction
Total	258 (100.00)	−0.53 (−0.88 to −0.18)	**0** **.** **004**	
Gender				0.081
Female	120 (46.51)	−0.84 (−1.32 to −0.37)	**<** **.** **001**	
Male	138 (53.49)	−0.22 (−0.74 to 0.30)	0.403	
Hypertension				0.600
No	193 (74.81)	−0.46 (−0.86 to −0.06)	**0** **.** **025**	
Yes	65 (25.19)	−0.70 (−1.53 to 0.13)	0.103	
Diabetes mellitus				0.973
No	223 (86.43)	−0.53 (−0.89 to −0.16)	**0** **.** **005**	
Yes	35 (13.57)	−0.51 (−1.78 to 0.77)	0.440	
Smoking				0.815
No	202 (78.29)	−0.50 (−0.91 to −0.10)	**0** **.** **016**	
Yes	56 (21.71)	−0.60 (−1.32 to 0.12)	0.108	
Drinking				0.993
No	170 (65.89)	−0.53 (−0.98 to −0.07)	**0** **.** **025**	
Yes	88 (34.11)	−0.52 (−1.08 to 0.04)	0.070	
PVC course				0.205
F-HR-PVC	151 (58.53)	−0.32 (−0.77 to 0.13)	0.163	
S-HR-PVC	41 (15.89)	−0.64 (−1.45 to 0.17)	0.132	
I-HR-PVC	66 (25.58)	−1.14 (−1.92 to −0.36)	**0** **.** **006**	
Origin type1				0.781
Right ventricle	159 (61.63)	−0.49 (−0.95 to −0.02)	**0** **.** **042**	
Left ventricle	99 (38.37)	−0.59 (−1.12 to −0.06)	**0** **.** **030**	
Origin type2				0.387
Outflow tract	173 (67.05)	−0.44 (−0.86 to −0.01)	**0** **.** **046**	
Non-outflow tract	85 (32.95)	−0.77 (−1.40 to −0.14)	**0** **.** **018**	
QRS duration				0.078
<150 ms	215 (83.33)	−0.42 (−0.80 to −0.03)	**0** **.** **034**	
≥150 ms	43 (16.67)	−1.22 (−2.11 to −0.33)	**0** **.** **011**	
Paired PVC				0.479
No	164 (63.57)	−0.43 (−0.87 to 0.01)	0.057	
Yes	94 (36.43)	−0.71 (−1.34 to −0.09)	**0** **.** **027**	
Interpolated PVC				0.751
No	169 (65.50)	−0.56 (−0.98 to −0.15)	**0** **.** **009**	
Yes	89 (34.50)	−0.43 (−1.12 to 0.26)	0.227	
NSVT				0.977
No	212 (82.17)	−0.52 (−0.90 to −0.15)	**0** **.** **007**	
Yes	46 (17.83)	−0.54 (−1.62 to 0.54)	0.336	
Symptomatic PVC				0.900
No	114 (44.19)	−0.43 (−1.03 to 0.17)	0.166	
Yes	144 (55.81)	−0.47 (−0.92 to −0.03)	**0** **.** **037**	

NSVT, non-sustained ventricular tachycardia.

Bold values indicate *P* < 0.05.

**Table 6:** Subgroup analysis of the relationship between GCS and LVEF.

Variables	*n* (%)	*β* (95%CI)	*P*	*P* for interaction
Total	258 (100.00)	−0.57 (−0.89 to −0.25)	**<** **.** **001**	
Gender				0.357
Female	120 (46.51)	−0.70 (−1.15 to −0.26)	**0** **.** **002**	
Male	138 (53.49)	−0.40 (−0.86 to 0.06)	0.094	
Hypertension				0.037
No	193 (74.81)	−0.39 (−0.75 to −0.02)	**0** **.** **039**	
Yes	65 (25.19)	−1.21 (−1.87 to −0.54)	**<** **.** **001**	
Diabetes mellitus				0.641
No	223 (86.43)	−0.55 (−0.88 to −0.21)	**0** **.** **001**	
Yes	35 (13.57)	−0.81 (−1.95 to 0.33)	0.175	
Smoking				0.694
No	202 (78.29)	−0.61 (−0.98 to −0.24)	**0** **.** **002**	
Yes	56 (21.71)	−0.46 (−1.11 to 0.18)	0.164	
Drinking				0.530
No	170 (65.89)	−0.50 (−0.90 to −0.09)	**0** **.** **017**	
Yes	88 (34.11)	−0.71 (−1.25 to −0.17)	**0** **.** **011**	
PVC course				0.080
F-HR-PVC	151 (58.53)	−0.31 (−0.74 to 0.11)	0.151	
S-HR-PVC	41 (15.89)	−1.00 (−1.71 to −0.28)	**0** **.** **009**	
I-HR-PVC	66 (25.58)	−1.11 (−1.75 to −0.46)	**0** **.** **001**	
Origin type1				0.676
Right ventricle	159 (61.63)	−0.64 (−1.07 to −0.21)	**0** **.** **004**	
Left ventricle	99 (38.37)	−0.50 (−0.97 to −0.03)	**0** **.** **041**	
Origin type2				0.567
Outflow tract	173 (67.05)	−0.52 (−0.91 to −0.12)	**0** **.** **012**	
Non-outflow tract	85 (32.95)	−0.71 (−1.26 to −0.16)	**0** **.** **013**	
QRS duration				0.003
<150 ms	215 (83.33)	−0.39 (−0.74 to −0.05)	**0** **.** **025**	
≥150 ms	43 (16.67)	−1.72 (−2.56 to −0.89)	**<** **.** **001**	
Paired PVC				0.572
No	164 (63.57)	−0.63 (−1.05 to −0.22)	**0** **.** **003**	
Yes	94 (36.43)	−0.43 (−0.98 to 0.11)	0.125	
Interpolated PVC				0.370
No	169 (65.50)	−0.50 (−0.88 to −0.12)	**0** **.** **011**	
Yes	89 (34.50)	−0.86 (−1.51 to −0.21)	**0** **.** **011**	
NSVT				0.590
No	212 (82.17)	−0.61 (−0.96 to −0.27)	**<** **.** **001**	
Yes	46 (17.83)	−0.36 (−1.32 to 0.60)	0.464	
Symptomatic PVC				0.443
No	114 (44.19)	−0.35 (−0.95 to 0.25)	0.259	
Yes	144 (55.81)	−0.62 (−0.98 to −0.25)	**0** **.** **001**	

NSVT, non-sustained ventricular tachycardia.

Bold values indicate *P* < 0.05.

## 4 Discussion

This study compared the baseline characteristics and key echocardiographic parameters, including GLS, GCS and LVEF, between patients with PVCs and healthy controls. The results demonstrated no significant difference in LVEF between the two groups, whereas both GLS and GCS were significantly different, suggesting that GLS and GCS may serve as more sensitive early indicators of PVCCM than LVEF. Therefore, we further utilized GLS and GCS to assess the left ventricular (LV) function and identify risk factors associated with PVCCM.

### 4.1 Possible mechanisms and background of PVCCM

Premature ventricular complexes (PVCs) are frequently observed in both individuals with and without structural heart disease. Although historically regarded as benign, accumulating evidence suggests that a high burden of PVCs may contribute to the development of PVC-induced cardiomyopathy, characterized by reversible left ventricular (LV) dysfunction (34, 35). The pathophysiological mechanisms underlying PVC-induced cardiomyopathy are multifactorial and include ventricular dyssynchrony, impaired calcium homeostasis, shortened coupling intervals leading to mechanical inefficiency, post-extrasystolic potentiation, autonomic imbalance, and mitochondrial dysfunction. Chronic high-burden PVCs can promote adverse left ventricular remodeling, ultimately resulting in reversible systolic dysfunction (3, 36–40). However, its diagnosis remains largely retrospective and exclusionary, lacking definitive prospective criteria. Furthermore, there is ongoing debate regarding the management of asymptomatic patients with a high PVC burden but preserved left ventricular ejection fraction (LVEF). This highlights the need for early functional markers—such as strain imaging by speckle tracking echocardiography—to detect subclinical myocardial dysfunction and guide timely intervention.

Despite the association between PVC burden and cardiomyopathy, population-based data have shown that PVC burden alone is not an independent predictor (*P* = 0.13) (41). This highlights the diagnostic challenge and supports the ASE/EACVI guideline recommendation to adopt a multiparametric approach incorporating advanced strain analysis (42). Beyond PVC burden, morphological heterogeneity may exert distinct pathophysiological effects. Paired PVCs, for example, aggravate ventricular dyssynchrony and calcium handling abnormalities compared with isolated beats (5), while interpolated PVCs, by occurring without compensatory pauses, alter preload and increase diastolic wall stress, thereby promoting fibrosis (5). Non-sustained ventricular tachycardia (NSVT), as an extension of consecutive PVCs, has been associated with more severe electromechanical dyssynchrony, impaired myocardial perfusion, and adverse metabolic remodeling (43). Advanced imaging further supports these differences: patients with NSVT demonstrate pronounced mechanical dyssynchrony and abnormal global longitudinal strain even in sinus rhythm, and cardiac MRI studies reveal a strong association between NSVT and myocardial fibrosis (5, 7). Clinically, this translates into divergent prognostic implications, with NSVT conferring higher risks of malignant arrhythmias, heart failure progression, and mortality, whereas high-burden PVCs are more closely linked to progressive LV dysfunction (7). These findings suggest that not only PVC burden but also morphological patterns may play a pivotal role in the pathogenesis and outcomes of PVCCM.

### 4.2 Association of GLS and GCS with PVCCM

Correlation analysis revealed multiple factors significantly associated with GLS and GCS deterioration, including PVC burden, asymptomatic PVC, and specific PVC morphologies such as interpolated PVC, paired PVC, and NSVT. Patients with these characteristics exhibited attenuated GLS and GCS magnitude, indicating impaired myocardial strain and suggesting that these factors may contribute to early myocardial dysfunction and the development of PVCCM. Many previous studies have suggested that premature beat load is negatively correlated with cardiac function, suggesting that premature beat load is one of the risk factors for PVC cardiomyopathy (44–46), which is consistent with our findings.

By employing various statistical methodologies, we confirmed significant negative correlations between LVEF and both GLS and GCS. As LVEF decreased, GLS and GCS values showed attenuated magnitude, indicating progressive deterioration in myocardial contractile function. LVEF measures the percentage of blood ejected from the left ventricle per cardiac cycle, whereas GLS evaluates longitudinal myocardial fiber contraction, and GCS assesses circumferential fiber shortening, offering complementary insights into myocardial mechanics and providing a more comprehensive assessment of LV function.

Compared to LVEF, GLS and GCS are more sensitive and earlier indicators of myocardial dysfunction. Even when LVEF remains within the normal range, GLS can detect subclinical myocardial impairment. In acute myocardial infarction (AMI) patients, prior studies have demonstrated that GLS can identify subclinical LV dysfunction even when LVEF is preserved, offering prognostic information beyond LVEF alone (19, 47). Furthermore, GLS has been established as an independent predictor of adverse cardiovascular outcomes. Studies in dilated cardiomyopathy (DCM) patients indicate that GLS is a stronger predictor of adverse events than LVEF, highlighting its value in risk stratification (19). Even in patients with LVEF ≤ 35%, both GLS and GCS provide additional prognostic information (20). In contrast, global radial strain (GRS) was not analyzed in this study due to its limited reliability in routine transthoracic echocardiography (48, 49). Moreover, GLS and GCS demonstrate superior reproducibility compared to LVEF, regardless of the operator's level of echocardiographic training (21). This suggests that GLS and GCS can provide consistent and reliable assessments across different clinical settings. Additionally, in patients undergoing cancer therapy, GLS has been employed for early detection of cardiotoxicity, allowing for intervention before significant LVEF deterioration occurs. This application underscores the utility of GLS in guiding clinical decisions to prevent irreversible cardiac damage (50).

### 4.3 Subgroup analysis of GLS, GCS, and LVEF

Further subgroup analysis revealed that the negative correlation between GLS, GCS, and LVEF holds significant clinical relevance in patients with hypertension and wide QRS. Previous studies have demonstrated that GLS and GCS are significantly reduced in hypertensive patients, indicating impaired myocardial contractility, even when LVEF remains within the normal range. This suggests that LVEF alone may not be sufficient to detect early myocardial dysfunction in these patients. Therefore, GLS and GCS can serve as more sensitive markers of subclinical myocardial impairment, facilitating early detection and intervention in hypertensive populations (51). Similarly, in patients with prolonged QRS duration, particularly those with left bundle branch block (LBBB), GLS and GCS reductions are associated with progressive LV dysfunction. These strain parameters can be utilized to predict further LV deterioration, aiding in treatment decision-making (52). While most studies support the clinical utility of GLS and GCS, their prognostic value may vary across specific patient populations, necessitating further investigation (53).

### 4.4 Study strengths and limitations

This study has several methodological strengths. This study has several methodological strengths. It uniquely integrates conventional echocardiographic assessment with speckle-tracking analysis to quantitatively evaluate myocardial deformation in patients with frequent premature ventricular contractions (PVCs). By applying global longitudinal strain (GLS) and global circumferential strain (GCS), this study provides a more refined approach to detecting early myocardial dysfunction. The design also incorporated a broad spectrum of PVC characteristics—including asymptomatic PVCs, interpolated PVCs, paired PVCs, and non-sustained ventricular tachycardia (NSVT)—to assess their potential impact on myocardial strain, which remains underexplored in existing literature. These aspects together contribute to a more comprehensive evaluation of strain-based risk factors for PVC-induced cardiomyopathy (PVCCM).

However, several limitations should be acknowledged. First, the cross-sectional design restricts the ability to infer causality between PVC burden and myocardial dysfunction. The associations identified in this study should be interpreted as correlational rather than causal, and longitudinal studies are warranted to confirm the temporal and predictive relationships of strain parameters with the development of PVCCM. In particular, it remains unclear whether complex PVCs—especially NSVT—represent early myocardial impairment due to an underlying cardiomyopathy or are primarily induced by frequent PVCs. This distinction is clinically important given the relatively high prevalence of NSVT observed in our cohort compared to typical populations. Second, the relatively small sample size may reduce the generalizability of our findings and increase the potential influence of outliers. Future studies with larger, more diverse cohorts are necessary. Third, although speckle-tracking echocardiography (STE) is less operator-dependent than conventional LVEF assessment, image quality and manual selection of optimal cardiac cycles remain critical. These factors may introduce observer variability. The implementation of standardized imaging protocols and automated strain analysis could enhance reproducibility. A limitation of this study is that only intraobserver variability was assessed, whereas interobserver reproducibility was not evaluated. Fourth, this study did not account for potential confounding variables such as coronary artery disease, obstructive sleep apnea, or the use of medications like beta-blockers and antiarrhythmics, all of which may influence myocardial strain and PVC burden. Fifth, selection bias may exist due to the exclusion of patients with structural heart disease, prior electrophysiological interventions, or ongoing antiarrhythmic therapy. These criteria, while necessary for defining a homogeneous study population, may limit the generalizability of our findings.

## 5 Conclusion

This study highlights GLS and GCS as more sensitive markers than LVEF for detecting myocardial dysfunction associated with frequent PVCs. The PVC burden, asymptomatic PVC, PVC with wide QRS, and the presence of specific PVC morphologies (including paired PVC, interpolated PVC, and NSVT) were observed in association with attenuated GLS and/or GCS magnitude, suggestive of impaired myocardial strain. We observed significant negative correlations between GLS, GCS, and LVEF, particularly in patients with hypertension, wide QRS, and asymptomatic PVC. Future research should focus on larger prospective studies to further validate the utility of GLS and GCS in risk stratification and their potential implications for early management of PVCCM.

## Data Availability

The raw data supporting the conclusions of this article will be made available by the authors, without undue reservation.
