# Supplementary material for: Speckle tracking technology and investigation of risk factors for premature ventricular contraction-induced cardiomyopathy
Source: Front Cardiovasc Med. 2025 Sep 30;12:1675906. doi: 10.3389/fcvm.2025.1675906 (PMC12518328; doi:10.3389/fcvm.2025.1675906)
Supplement: Supplementary file 1 [file Table1.pdf]

Supplementary Table 1. Multicollinearity diagnostics for covariates included in the regression models

| Variable | VIF | Condition Index |
|---|---|---|
| LVEF | 1.07 | – |
| Gender | 1.05 | – |
| Age | 1.08 | – |
| BMI | 1.15 | – |
| PVC | 1.05 | – |
| QRS.group | 1.03 | – |
| Paired PVC | 1.05 | – |
| Interpolated | 1.03 | – |
| Symptom | 1.16 | – |

Interpretation: All VIFs <2, indicating no evidence of multicollinearity. Condition indices were not elevated, confirming absence of collinearity problems
