# Supplementary material for: Speckle tracking technology and investigation of risk factors for premature ventricular contraction-induced cardiomyopathy
Source: Front Cardiovasc Med. 2025 Sep 30;12:1675906. doi: 10.3389/fcvm.2025.1675906 (PMC12518328; doi:10.3389/fcvm.2025.1675906)
Supplement: Supplementary file 2 [file Table2.pdf]

Supplementary Table 2. The relationship between quantitative data and GLS and GCS

Variable	GLS		GCS	
	r	P	r	P
age	0.178	0.004	0.096	0.125
BMI	0.192	0.002	0.081	0.195
PVC burden	0.186	0.003	0.132	0.035
QT interval	0.069	0.270	0.032	0.603
QRS duration	0.117	0.060	0.074	0.238
coupling interval	0.073	0.244	0.073	0.244
compensatory pause	0.052	0.408	0.029	0.643
coupling interval index	0.089	0.155	0.058	0.356
compensatory pause index	0.055	0.379	0.070	0.261
