# Supplementary material for: Speckle tracking technology and investigation of risk factors for premature ventricular contraction-induced cardiomyopathy
Source: Front Cardiovasc Med. 2025 Sep 30;12:1675906. doi: 10.3389/fcvm.2025.1675906 (PMC12518328; doi:10.3389/fcvm.2025.1675906)
Supplement: Supplementary file 3 [file Table3.pdf]

Supplementary Table 3. GLS, GCS, and their associations with baseline and electrocardiographic parameters

Variable	n (%)	GLS, Mean ± SD	Statistic	P	GCS, Mean ± SD	Statistic	P
Total	258 (100)	-14.78 ± 1.88			-14.80 ± 2.04		
Gender			t = 1.28	0.202		t = -1.10	0.271
Female	120 (46.51)	-14.62 ± 2.02			-14.95 ± 2.16		
Male	138 (53.49)	-14.92 ± 1.74			-14.67 ± 1.93		
Hypertension			t = -3.24	0.001		t = -0.74	0.462
No	193 (74.81)	-14.99 ± 1.91			-14.86 ± 2.09		
Yes	65 (25.19)	-14.14 ± 1.64			-14.64 ± 1.91		
Diabetes mellitus			t = -1.32	0.190		t = -0.32	0.753
No	223 (86.43)	-14.84 ± 1.92			-14.82 ± 2.09		
Yes	35 (13.57)	-14.39 ± 1.59			-14.70 ± 1.74		
Smoking			t = -2.04	0.043		t = -1.10	0.270
No	202 (78.29)	-14.90 ± 1.83			-14.87 ± 1.98		
Yes	56 (21.71)	-14.33 ± 2.01			-14.53 ± 2.27		
Drinking			t = -0.53	0.597		t = 0.06	0.953
No	170 (65.89)	-14.82 ± 1.77			-14.80 ± 2.01		
Yes	88 (34.11)	-14.69 ± 2.08			-14.81 ± 2.12		
PVC course			F = 1.44	0.238		F = 0.83	0.439
F-HR-PVC	151 (58.53)	-14.88 ± 1.99			-14.91 ± 2.12		
S-HR-PVC	41 (15.89)	-14.96 ± 1.81			-14.46 ± 1.94		
I-HR-PVC	66 (25.58)	-14.44 ± 1.64			-14.75 ± 1.94		
Origin type1			t = 0.15	0.879		t = -1.01	0.312
Right ventricle	159 (61.63)	-14.76 ± 1.90			-14.90 ± 2.02		
Left ventricle	99 (38.37)	-14.80 ± 1.85			-14.64 ± 2.08		
Origin type2			t = -1.33	0.186		t = -1.05	0.297
Outflow tract	173 (67.05)	-14.89 ± 1.90			-14.89 ± 2.02		
Non-outflow tract	85 (32.95)	-14.56 ± 1.83			-14.61 ± 2.08		
QRS duration			t = -2.57	0.011		t = -1.86	0.064
<150ms	215 (83.33)	-14.91 ± 1.82			-14.91 ± 2.04		
≥150ms	43 (16.67)	-14.11 ± 2.03			-14.27 ± 1.99		
Paired PVC			t = -3.08	0.002		t = -3.60	<.001
No	164 (63.57)	-15.05 ± 1.95			-15.14 ± 2.04		
Yes	94 (36.43)	-14.31 ± 1.66			-14.21 ± 1.92		
Interpolated PVC			t=-2.44	0.016		t=-2.81	0.005
No	169 (65.50)	-14.97 ± 2.00			-15.04 ± 2.20		
Yes	89 (34.50)	-14.42 ± 1.57			-14.36 ± 1.63		
NSVT			t = -2.75	0.006		t = -3.10	0.002
No	212 (82.17)	-14.93 ± 1.89			-14.98 ± 2.04		
Yes	46 (17.83)	-14.10 ± 1.66			-13.97 ± 1.88		
Symptomatic PVC			t = 3.79	<.001		t=2.52	0.012
No	114 (44.19)	-14.29 ± 1.77			-14.45 ± 1.77		

Variable	n (%)	GLS, Mean ± SD	Statistic	P	GCS, Mean ± SD	Statistic	P
Yes	144 (55.81)	-15.16 ± 1.88			-15.08 ± 2.21		

SD: standard deviation

t: t-test, F: ANOVA
