# Supplementary material for: Speckle tracking technology and investigation of risk factors for premature ventricular contraction-induced cardiomyopathy
Source: Front Cardiovasc Med. 2025 Sep 30;12:1675906. doi: 10.3389/fcvm.2025.1675906 (PMC12518328; doi:10.3389/fcvm.2025.1675906)
Supplement: Supplementary file 4 [file Table4.pdf]

Supplementary Table 4. Independent Correlates of GLS

Variables	single factor					multi-factor				
	β	SE	t	P	β (95%CI)	β	SE	t	P	β (95%CI)
Gender										
Female					0.00 (Reference)					
Male	-0.30	0.23	-1.28	0.202	-0.30 (-0.76 ~ 0.16)					
Hypertension										
No					0.00 (Reference)					0.00 (Reference)
Yes	0.86	0.26	3.24	**0.001**	0.86 (0.34 ~ 1.38)	0.43	0.28	1.53	0.127	0.43 (-0.12 ~ 0.98)
Diabetes mellitus										
No					0.00 (Reference)					
Yes	0.45	0.34	1.32	0.190	0.45 (-0.22 ~ 1.12)					
Drinking										
No					0.00 (Reference)					
Yes	0.13	0.25	0.53	0.597	0.13 (-0.35 ~ 0.62)					
Smoking										
No					0.00 (Reference)					0.00 (Reference)
Yes	0.57	0.28	2.04	**0.043**	0.57 (0.02 ~ 1.13)	0.39	0.26	1.48	0.140	0.39 (-0.12 ~ 0.90)
Symptomatic PVC										
No					0.00 (Reference)					0.00 (Reference)
Yes	-0.87	0.23	-3.79	**<.001**	-0.87 (-1.32 ~ -0.42)	-0.99	0.21	-4.64	**<.001**	-0.99 (-1.41 ~ -0.57)
PVC course										
F-HR-PVC					0.00 (Reference)					
S-HR-PVC	-0.08	0.33	-0.24	0.810	-0.08 (-0.73 ~ 0.57)					
I-HR-PVC	0.43	0.28	1.56	0.119	0.43 (-0.11 ~ 0.98)					
Origin type1										
right ventricle					0.00 (Reference)					
left ventricle	-0.04	0.24	-0.15	0.879	-0.04 (-0.51 ~ 0.44)					
Origin type2										
Outflow tract					0.00 (Reference)					
Non-outflow tract	0.33	0.25	1.33	0.186	0.33 (-0.16 ~ 0.82)					
QRS duration										

Variables	single factor					multi-factor				
	β	SE	t	P	β (95%CI)	β	SE	t	P	β (95%CI)
<150ms					0.00 (Reference)					0.00 (Reference)
≥150ms	0.80	0.31	2.57	**0.011**	0.80 (0.19 ~ 1.41)	0.97	0.28	3.43	**<.001**	0.97 (0.42 ~ 1.52)
Paired PVC										
No					0.00 (Reference)					0.00 (Reference)
Yes	0.74	0.24	3.08	**0.002**	0.74 (0.27 ~ 1.20)	0.71	0.22	3.20	**0.002**	0.71 (0.27 ~ 1.14)
Interpolated PVC										
No					0.00 (Reference)					0.00 (Reference)
Yes	0.55	0.24	2.26	**0.025**	0.55 (0.07 ~ 1.03)	0.67	0.22	3.03	**0.003**	0.67 (0.24 ~ 1.11)
NSVT										
No					0.00 (Reference)					
Yes	0.83	0.30	2.75	**0.006**	0.83 (0.24 ~ 1.42)					
Age	0.02	0.01	2.90	**0.004**	0.02 (0.01 ~ 0.04)	0.01	0.01	1.67	0.096	0.01 (-0.00 ~ 0.03)
BMI	0.10	0.03	3.13	**0.002**	0.10 (0.04 ~ 0.17)					
PVC burden	0.06	0.02	3.02	**0.003**	0.06 (0.02 ~ 0.11)	0.04	0.02	2.09	**0.037**	0.04 (0.01 ~ 0.08)
QTc	-0.01	0.00	-1.11	0.270	-0.01 (-0.01 ~ 0.00)					
QRS	0.02	0.01	1.89	0.060	0.02 (-0.00 ~ 0.04)					
Coupling interval	-0.00	0.00	-1.17	0.244	-0.00 (-0.01 ~ 0.00)					
Compensatory pause	0.00	0.00	0.83	0.408	0.00 (-0.00 ~ 0.00)					
Coupling interval index	-2.92	2.06	-1.42	0.157	-2.92 (-6.96 ~ 1.11)					
Compensatory pause index	1.18	1.39	0.85	0.395	1.18 (-1.54 ~ 3.91)					
LVEF	-0.06	0.02	-2.95	**0.004**	-0.06 (-0.10 ~ -0.02)	-0.04	0.02	-1.99	**0.048**	-0.04 (-0.08 ~ -0.01)

CI: Confidence Interval
