# Supplementary material for: Speckle tracking technology and investigation of risk factors for premature ventricular contraction-induced cardiomyopathy
Source: Front Cardiovasc Med. 2025 Sep 30;12:1675906. doi: 10.3389/fcvm.2025.1675906 (PMC12518328; doi:10.3389/fcvm.2025.1675906)
Supplement: Supplementary file 5 [file Table5.pdf]

Supplementary Table 5. Independent Correlates of GCS

Variables	single factor					multi-factor				
	β	SE	t	*P*	β (95%CI)	β	SE	t	*P*	β (95%CI)
Gender										
female					0.00 (Reference)					
male	0.28	0.26	1.10	0.271	0.28 (-0.22 ~ 0.78)					
Hypertension										
No					0.00 (Reference)					
Yes	0.22	0.29	0.74	0.462	0.22 (-0.36 ~ 0.79)					
Diabetes mellitus										
No					0.00 (Reference)					
Yes	0.12	0.37	0.32	0.753	0.12 (-0.61 ~ 0.85)					
Drinking										
No					0.00 (Reference)					
Yes	-0.02	0.27	-0.06	0.953	-0.02 (-0.54 ~ 0.51)					
Smoking										
No					0.00 (Reference)					
Yes	0.34	0.31	1.10	0.270	0.34 (-0.26 ~ 0.95)					
symptomatic PVC										
No					0.00 (Reference)					0.00 (Reference)
Yes	-0.62	0.25	-2.45	**0.015**	-0.62 (-1.12 ~ -0.13)	-0.70	0.24	-2.89	**0.004**	-0.70 (-1.18 ~ -0.23)
PVC course										
F-HR-PVC					0.00 (Reference)					
S-HR-PVC	0.46	0.36	1.27	0.206	0.46 (-0.25 ~ 1.16)					
I-HR-PVC	0.16	0.30	0.54	0.592	0.16 (-0.43 ~ 0.75)					
Origin type1										
right ventricle					0.00 (Reference)					
left ventricle	0.26	0.26	1.01	0.312	0.26 (-0.25 ~ 0.78)					
Origin type2										
Outflow tract					0.00 (Reference)					
Non-outflow tract	0.28	0.27	1.05	0.297	0.28 (-0.25 ~ 0.81)					
QRS duration										
<150ms					0.00 (Reference)					0.00 (Reference)
≥150ms	0.63	0.34	1.86	0.064	0.63 (-0.03 ~ 1.30)	0.84	0.32	2.64	**0.009**	0.84 (0.22 ~ 1.46)
Paired PVC										
No					0.00 (Reference)					0.00 (Reference)
Yes	0.93	0.26	3.60	**<.001**	0.93 (0.43 ~ 1.44)	0.95	0.25	3.86	**<.001**	0.95 (0.47 ~ 1.44)
Interpolated PVC										
No					0.00 (Reference)					0.00 (Reference)
Yes	0.68	0.26	2.57	**0.011**	0.68 (0.16 ~ 1.20)	0.76	0.25	3.04	**0.003**	0.76 (0.27 ~ 1.25)
NSVT										
No					0.00 (Reference)					

Variables	single factor					multi-factor				
	β	SE	t	*P*	β (95%CI)	β	SE	t	*P*	β (95%CI)
Yes	1.01	0.33	3.10	**0.002**	1.01 (0.37 ~ 1.66)					
Age	0.01	0.01	1.54	0.125	0.01 (-0.00 ~ 0.03)					
BMI	0.05	0.04	1.30	0.195	0.05 (-0.02 ~ 0.12)					
PVC burden	0.05	0.02	2.13	**0.035**	0.05 (0.01 ~ 0.10)					
QTc	-0.00	0.01	-0.52	0.603	-0.00 (-0.01 ~ 0.01)					
QRS	0.01	0.01	1.18	0.238	0.01 (-0.01 ~ 0.03)					
Coupling interval	-0.00	0.00	-1.17	0.244	-0.00 (-0.01 ~ 0.00)					
Compensatory pause	0.00	0.00	0.46	0.643	0.00 (-0.00 ~ 0.00)					
Coupling interval index	-2.06	2.24	-0.92	0.359	-2.06 (-6.46 ~ 2.34)					
Compensatory pause index	1.64	1.51	1.08	0.279	1.64 (-1.32 ~ 4.60)					
LVEF	-0.08	0.02	-3.50	**<.001**	-0.08 (-0.12 ~ -0.04)	-0.07	0.02	-3.12	**0.002**	-0.07 (-0.11 ~ -0.03)

CI: Confidence Interval
